# Periodontal Status and Risk Factors among Adults of Sebha City (Libya)

**DOI:** 10.1155/2012/787502

**Published:** 2012-11-14

**Authors:** Syed Wali Peeran, A. J. A. Ranjith Singh, G. Alagamuthu, Syed Ali Peeran, P. G. Naveen Kumar

**Affiliations:** ^1^Department of Periodontology and Oral Implantology, Faculty of Dentistry, Sebha University, Sebha, Libya; ^2^Department of Advanced Zoology and Biotechnology, Sri Paramakalyani College, Alwarkurichi, India; ^3^Department of Chemistry, Sri Paramakalyani College, Alwarkurichi, India; ^4^Faculty of Dentistry, Gezan University, Gezan, Saudi Arabia; ^5^Department of Community Dentistry, Faculty of Dentistry, Sebha University, Sebha, Libya

## Abstract

The present study was aimed at assessing the periodontal status and risk factors like age, gender, tooth brushing habit, and smoking among the adult population of Sebha city, Libya. 452 adults, aged 35–54 years, comprised the study sample. 266 (58.84%) were females and 186 (41.15%) were males. Data was collected by interview and clinical examination using CPI of CPITN index. Chi-square test and ANOVA were used for statistical analysis at 5% level of significance. Results indicate that 76.32% used toothbrush and paste; 8.84% were current smokers and were all males. Majority, 52.65% were, detected with shallow pockets followed by 30.08% with calculus, 12.17% had deep pockets, 3.31% had bleeding, and only 1.33% were healthy. Age, gender, current smoking status and frequency of tooth brushing showed statistically significant difference with CPI codes. Health professionals can utilize this data to identify individuals at risk and to target population level interventions.

## 1. Introduction

Available data indicates that the prevalence of periodontal disease is very high in several African countries affecting all age groups [1]. Libya is one of the North African country in the Mediterranean region and Sebha is the largest city situated in the south of the Libyan Arab Jamahiriya and located in the middle of Libyan desert. People come to Sebha from the various urban areas surrounding it and therefore it has a heterogeneous society [2]. Information on the oral health status of the Sebha population is scarce. To date, except for the report of Hassan [2] and Leous [3], there is a paucity in publication concerning the oral health in general and the prevalence and severity of periodontal diseases in particular.

Since 1993, WHO began collecting data on periodontal status from different countries. This data is stored in the WHO Global Oral Data Bank (GODB) [4]. The periodontal country profile for Libya is mentioned in the WHO data base, which is an extract of the 1982-1983 pathfinder study [3], conducted to assess the oral health situation in Socialist People's Libyan Arab Jamahiriya. It was conducted on 849 children and 269 adults from 14 sites of the following localities namely Tripoli, Sebha, Benghazi, Zwara, Ajelat, and Kaddah. CPITN index was used to assess the periodontal disease and its results indicate that the periodontal disease is present from a very young age. There was increased tooth loss by the age of 55–64 years. High prevalence of periodontal disease was the main cause of tooth loss in adulthood. Among the 35–44 years old, there was none with healthy periodontium and bleeding. 13% had calculus, 53% had shallow pockets, and 34% deep pockets. The average number of sextants with bleeding and higher score was 5.7. Ever since the completion of this study, two decades have passed and there is no new data which has been updated.

Age [5, 6], gender [7, 8], toothbrushing [9, 10], and smoking [7, 11–13] are among the important risk factors for periodontal disease. Information of the periodontal status and additional information of risk factors can help the dental professional, to identify people in the high risk for periodontal disease and undertake strategic planning for a preventive and therapeutic treatment program.

 Against this background, the present study has been designed to assess the periodontal status and risk factors like age, gender, frequency of toothbrushing, and smoking of the adult population in Sebha city, Libya.

## 2. Material and Methods

 This study is a part of the extended project which was undertaken to evaluate the periodontal status and its determinants on a large scale amongst the population of Sebha (details are published elsewhere). Here,data of the adult population aged 35–54 years old is presented. One WHO, index age group of 35–44 years and other group of 45–54 years which are representative of adult population was considered. For sampling, the WHO guidelines (1997) [14] were followed. 4 sites were identified and 50 subjects per site for each of the 2 age groups were selected. An additional 10 subjects each were added to compensate for any non-participation and hence the sample was 480 (4 × 2 × 60). The survey was conducted at the Primary Health Centre (PHC) in Al-Jadeed, Al-Mahdia, Gurda, and Sukkara. The medical officer posted at the PHC was responsible to collect the participants for the study in the respective areas. People from household, offices, and those visiting the PHC comprised the study sample. All those who agreed to voluntarily participate were recruited for the survey. The participant was first interviewed to collect general information and data about the oral hygiene practices and smoking habits. The participant was asked simple questions about the oral hygiene aids used (toothbrush and others), use of toothpaste (yes, no), frequency of toothbrushing (once, twice, >twice), current smoker (yes, no), and frequency of smoking (5, 6–10, 11–20, >20/per day). After the interview, the participant underwent a clinical examination. ADA type 3 examination procedure was followed. CPI of the CPITN was used to record the periodontal status [14]. The main outcome measures of CPI are presence of gingival bleeding on gentle probing, dental calculus, and probing periodontal pocket depth (PPD): 4-5 or ≥6 mm. The dentition was divided into six sextants. The index teeth were 16, 17, 11, 26, 27, and 36, 37, 31, 46, 47. When two index teeth were considered in a sextant, the tooth with the highest score was recorded. The overall CPI score of the participant represented the value of the highest recorded code for that individual. 

The examination was performed by a single, trained, calibrated examiner assisted by a recording clerk, using a CPI probe and mouth mirror. During calibration 10% of the sample was reexamined and kappa value of 0.8 was obtained which indicates a good intra-examiner consistency. All the subjects were well educated and signed the informed consent form. The ethical board, scientific committee of the Faculty of dentistry, Sebha University, Sebha, Libya approved the protocol of the study.

Statistical analysis was done with the help of a statistical package for social sciences (SPSS version 16). Mean standard deviation and percentages were calculated. Chi-square test and ANOVA were used to compare between the groups at 5% level of significance. The variables were age, gender, frequency of toothbrushing, and smoking habit, which were compared with the CPI codes. 

## 3. Results

The sample size calculated was 480, but the survey could be completed among 452 people, of which 266 (58.84%) were females and 186 (41.16%) were males. 249 (55.08%) were in the age group of 35–44 years and 203 (44.92%) in 45–54 years, respectively.

Overall, 344 (76.15%) reported use of toothbrush and paste, 45 (9.96%) used finger, and 63 (13.93%) used siwak. 225 (49.78%) brushed once daily, 96 (21.23%) brushed twice, and 21 (4.64%) brushed more than twice. 40 (8.84%) were current smokers and were all males. 19 (4.2%) reported to have smoked 5–10 cigarettes, while 12 (2.65%) smoked 10–20/per day and 9 (1.95%) smoked more than 20.

**Figure 1:**
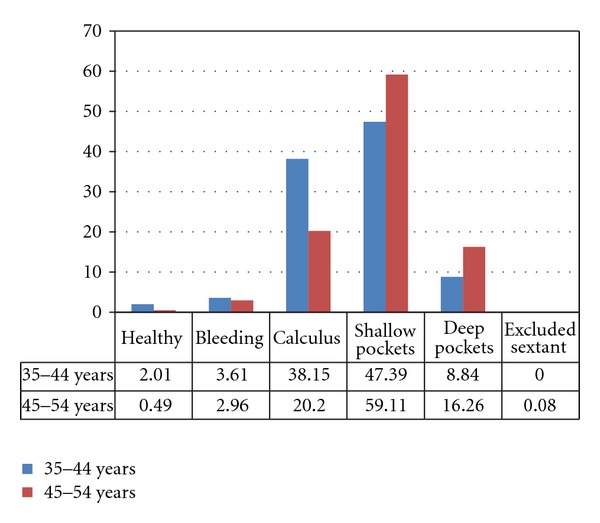
Percentage distribution of the subjects as per the CPI codes.

Majority, 52.65% (*n* = 238), were detected with shallow pockets (CPI code 3) followed by 30.08% (*n* = 136) with calculus (code 2), 12.17% (*n* = 55) had deep pockets (code 4), 3.31% (*n* = 15) had bleeding (code 1), and only 1.33% (*n* = 6) were healthy (code 0).  Figure 1 shows the distribution of the study sample according to the CPI codes. 

Table 1 shows the distribution of CPI codes in relation to the different variables considered in the study. Age, gender, current smoking status, and frequency of toothbrushing showed statistically significant difference with CPI codes. 

Table 2 shows that for 35–44 years, the mean number of sextant for calculus was significantly higher as compared to the shallow pockets which was higher in 45–54 years age group. 

The mean (SD) CPI score for the entire sample was 2.71 (0.77). The mean CPI score was 2.57 (0.79) and 2.89 (0.72) for 35–44 years and for 45–54 years, respectively. 

## 4. Discussion

The present study is the first population-based survey among adult population in Sebha in the recent times after the pathfinder survey conducted in 1982-1983 [3]. Thorough training and calibration of the examiner ensured reliable recording of the CPI (community periodontal index). The major advantages of the CPI are simplicity, speed, reproducibility, and international uniformity [15]. Although this index has certain shortcomings when used as a stand-alone means of assessing the extent and severity of periodontal disease [16] it has been widely used for descriptive periodontal epidemiologic studies. In addition, the CPI data has been used for surveillance of periodontal health at country and intercountry levels [15].

**Figure 2:**
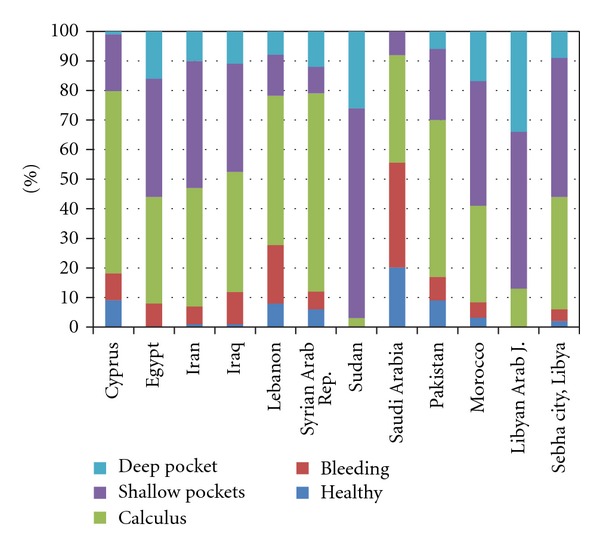
Overview of periodontal status of 35–44 year old from different EMRO countries. Data obtained from WHO, Global oral data bank. Sebha city, Libya-present study.

For the age group 35–44 years, the most frequently observed condition was shallow pockets of 4-5 mm among 47.39% followed by calculus in 38.15%. Deep pockets of more than 6 mm were found in 8.84% of the subjects. Bleeding was found in only 3.61% of the population surveyed. Only 2.01% had healthy periodontium.  Figure 2 shows the comparison of periodontal status for 35–44 years old from EMRO countries with the present study [4]. For the countries like Sudan, Iran, Egypt, Morocco, and Libya shallow pockets (code 3) was the highest recorded CPI score. However, calculus (code 2) as the highest score was reported for countries like Syrian Arab Rep., Cyprus, Pakistan, Lebanon, Saudi Arabia, and Iraq. The highest percentage of healthy periodontium (code 0) was reported for Saudi Arabia (20%). 34% Libyans and 26% Sudanese were among the ones to have high percentage of deep pockets (code 4). 

In a recent study among Sudanese [17], 36.1% had healthy periodontal tissues, 10.9% bleeding, 42.0% calculus, 8.5% 4-5 mm periodontal pocketing, 0.7% periodontal pocketing of ≥6 mm, and 1.8% excluded sextants. The Sudanese have better periodontal health as compared to the present study population.

When compared with the 1982-1983 pathfinder survey in Libya [3], a high percentage of people had shallow pockets and deep pockets versus the present study where percentage of shallow pockets and calculus was more. Even after 20 years, there is no improvement in the percentage of healthy periodontium (0% versus 1.33% in the present study) but the prevalence of severe form of periodontitis (deep pockets >6 mm) has been reduced from 34% to 12% in the present study.

It is widely assumed that symptoms of periodontal diseases escalates with age [6]. In the present study, percentage of subjects with healthy periodontium, bleeding, and calculus was comparatively more in the 35–44 years age group, while signs of periodontal destruction, that is, shallow pockets, and deep pockets were on a higher side among 45–54 years old (Figure 1).

In the present study, there was a significant statistical difference observed between the CPI codes and gender. The percentage calculation showed that the percentage of healthy periodontium and bleeding were higher among females and percentage of deep pockets were more prevalent among males. The percentage of calculus and shallow pockets were similar among the males and females. Studies indicate that prevalence of periodontal disease was slightly lower in females [18]. This may be because the females give more importance to aesthetics and have the habit of toothbrushing and seeking dental care regularly. 

Smoking can cause a number of changes within the periodontium which can predispose an individual to the progression of periodontal disease [19]. In a study, 5.5% of smokers had healthy periodontium compared to 44.2% among non-smokers and the periodontal condition of the smokers was very poor compared with that of non-smokers and this difference was statistically significant [19]. Literature [11] indicates that predominantly males, due to their smoking habit, have shown more susceptibility to periodontal diseases. The smokers in the present study were all males; significant difference in periodontal status was observed with the current smoking status, but when the data was stratified according to the frequency of smoking no statistically significant difference was found with the CPI codes. 

In the present study, 21.23% people brushed twice daily in comparison to 53% Sudanese [17] and 32% Chinese [20]. The frequency of toothbrushing was significantly associated with CPI codes in this study. Lang et al. [21] showed that frequent tooth brushing and flossing as well as regular dental attendance were found to be significantly associated with lower plaque, gingivitis, and calculus scores.

13.93% used siwak for cleaning their teeth in the present study. Studies have reported that in Saudi Arabia, 65% urban population used siwak on a daily basis [22]. Miswak (siwak) is known to have antibacterial effect on pathogens causing periodontal disease and caries. 

## 5. Conclusion

This study shows that more than half of the adult population in Sebha are detected with the signs of destructive periodontitis. If this trend continues, in the coming years the severity of periodontal disease is bound to increase enormously. In view of the potential association of periodontal disease with systemic disease (atherosclerosis, myocardial infarction, stroke, pneumonia, diabetes mellitus, and pregnancy) [23], this current health situation is of public health significance especially among adults and needs urgent attention. Hence, the study results can be utilized to identify individuals at risk of periodontal disease and to target population level interventions.

## Figures and Tables

**Table 1:** Number of subjects as per the CPI codes for different variables.

	Variable	Healthy	Bleeding	Calculus	Shallow pockets	Deep pockets	Chi-sqaure test
		(code 0)	(code 1)	(code 2)	(code 3)	(code 4)	(*P* value)
Age (in years)	35–44	5	9	95	118	22	22.05 (*P* = 0.0001)*
	45–54	1	6	41	120	33	

Gender	Male	0	3	54	98	31	14.42 (*P* = 0.01)*
	Female	6	12	82	140	24	

Frequency of toothbrushing	None	2	2	18	65	21	42.01 (*P* = 0.0001)*
	Once	0	9	69	127	20	
	Twice	4	4	41	35	12	
	>Twice	0	0	8	11	2	

Current smoking status	Yes	0	0	4	25	11	17.11 (*P* = 0.001)*
	No	6	15	132	213	44	

Frequency of smoking	0	6	15	132	213	44	18.28 (*P* = 0.2)
	5–10	0	0	2	12	5	
	10–20	0	0	2	7	3	
	>20	0	0	0	6	3	

*Statistically significant.

**Table 2:** Mean number (SD) of sextants affected by periodontal conditions in various age groups.

Age Group	Code 0*	Code 1**	Code 2***	Code 3^#^	Code 4^##^
35–44 yrs	1.36 (1.45)	0.55 (1.02)	2.53 (1.66)	1.30 (1.57)	0.18 (0.69)
45–54 yrs	1.33 (1.29)	0.28 (0.74)	1.84 (1.55)	2.04 (1.67)	0.38 (0.96)

ANOVA: F* = 0.09, *P* = 0.7632; F** = 9.39, *P* = 0.0023; F*** = 20.29, *P* < 0.0001; *F* ^#^ = 23.23, *P* < 0.0001; *F* ^##^ = 6.64, *P* = 0.0103. SD: Standard deviation.
